# Efficacy and safety of TACE combined with lenvatinib and PD‐1 inhibitors for unresectable recurrent HCC: A multicenter, retrospective study

**DOI:** 10.1002/cam4.5880

**Published:** 2023-03-31

**Authors:** Wei‐Jun Wang, Zong‐Han Liu, Kang Wang, Hong‐Ming Yu, Yu‐Qiang Cheng, Yan‐Jun Xiang, Jin‐Kai Feng, Li‐Ping Zhou, Hong‐Kun Zhou, Wei‐Wei Pan, Wei‐Xing Guo, Jie Shi, Shu‐Qun Cheng

**Affiliations:** ^1^ Department of Hepatic Surgery VI, Eastern Hepatobiliary Surgery Hospital Second Military Medical University Shanghai China; ^2^ Department of Hepatobiliary Surgery The First Affiliated Hospital of Wenzhou Medical University Wenzhou China; ^3^ The First Hospital of Jiaxing Affiliated Hospital of Jiaxing University Jiaxing University Jiaxing China; ^4^ Department of Cell Biology, College of Medicine Jiaxing University 118 Jiahang Road Jiaxing China; ^5^ G60 STI Valley Industry & Innovation Institute, Jiaxing University, Jiaxing University Jiaxing China

**Keywords:** combination therapy, hepatocellular carcinoma (HCC), lenvatinib, programmed death‐1, recurrent, transarterial chemoembolization (TACE)

## Abstract

**Background:**

There is no consensus on the optimal regimen for unresectable recurrent hepatocellular carcinoma (HCC), so this retrospective study aimed to evaluate the efficacy and safety of transarterial chemoembolization (TACE) combined with lenvatinib and PD‐1 inhibitors (T‐L‐P) versus TACE combined with lenvatinib (T‐L) or TACE alone.

**Method:**

Data were collected from 204 patients with unresectable recurrent HCC who received T‐L‐P, T‐L, or TACE alone at three medical centers from January, 2019 to December, 2020 for analysis. The survival outcomes, tumor response, and adverse events were compared between three groups, and risk factors were further investigated.

**Results:**

The median overall survival in the T‐L‐P, T‐L, and TACE alone groups were not reached, 25.6, and 15.7 months, respectively (*p* < 0.001). The median progression‐free survival in the T‐L‐P, T‐L, and TACE alone groups were 24.1, 17.3, and 13.7 months, respectively (*p* < 0.001). The best objective response rate in the T‐L‐P, T‐L, and TACE alone groups were 70.4%, 48.9%, and 42.5%, respectively. The best disease control rate in the T‐L‐P, T‐L, and TACE alone groups were 100.0%, 97.8%, and 87.5%, respectively. There was no significant difference between the T‐L‐P and T‐L groups for Grade 3/4 adverse events.

**Conclusion:**

T‐L‐P regimen was safe and superior to T‐L or TACE alone in improving survival for unresectable recurrent HCC patients.

## Graphical abstract

Transarterial chemoembolization (TACE) combined with Lenvatinib and PD‐1 inhibitors may have a better survival benefit and a manageable safety profile in patients with unresectable recurrent HCC compared with TACE plus Lenvatinib and TACE alone. This strategy has brought light to patients with unresectable recurrent HCC.

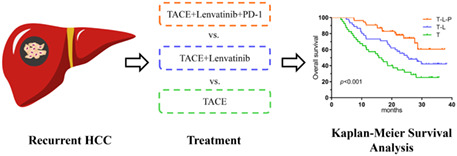

## 1 INTRODUCTION

Hepatocellular carcinoma (HCC) is the sixth most common malignancy worldwide and is one of the three major causes of cancer death.^1^, ^2^, ^3^ Although liver resection is recommended as the first‐line regimen, the high recurrence rate of 70% within 5 years is still a thorny issue confounding clinicians and patients.^4^ Furthermore, due to the limited function of residual liver or postoperative adhesion, re‐resection is not suitable for many patients, which creates an incentive to utilize other regimens.^5^, ^6^, ^7^

Transarterial chemoembolization (TACE) is usually adopted for most intrahepatic recurrent HCC because of multiple lesions, unfavorable location, and limited liver function.^8^, ^9^ However, the efficacy of TACE is unsatisfactory because of quick recurrence following this monotherapy.^10^ A prospective clinical study revealed that TACE in combination with apatinib significantly improved progression‐free survival (PFS) but not overall survival (OS) in patients with recurrent HCC.^11^ Besides, a retrospective controlled study demonstrated that the combination of TACE and lenvatinib significantly improved objective response rate (ORR) by nearly 36.6% over TACE monotherapy for unresectable HCC, but the treatment response remained limited and survival extension was poor.^12^

Immune checkpoint inhibitors have exhibited a promising efficacy recently.^13^ Although a phase III trial for anti‐PD‐1 monotherapy failed to meet the primary endpoint,^14^ a phase Ib study showed that combination of pembrolizumab and lenvatinib could achieve an ORR of 46.0% and a median OS of 22.0 months, which made an incredible impact on advanced HCC treatment.^15^ Besides, previous study showed that TACE enhanced the expression of programmed death‐1 (PD‐1) and programmed death‐ligand 1 (PD‐L1),^16^ which indicated the combination of TACE, lenvatinib and PD‐1 inhibitor might be conducive to a synergistic antitumor function. Our previous study showed that TACE combined with a PD‐1 inhibitor and lenvatinib had better efficacy in patients with intermediate‐stage primary hepatocellular carcinoma.^17^ However, no study has investigated whether this triple combination treatment could further improve the prognosis of patients with recurrent HCC.

Therefore, we enrolled patients with unresectable recurrent HCC who accepted the triple combination treatment (TACE, lenvatinib plus PD‐1 inhibitor (T‐L‐P)), double combination treatment (TACE and lenvatinib (T‐L)), or TACE monotherapy in this retrospective study to evaluate the efficacy and safety of these combination regimens for unresectable recurrent HCC.

## 2 PATIENTS AND METHODS

### 2.1 Patients

From January 2019 to December 2020, consecutive patients with recurrent HCC who underwent T‐L‐P, T‐L or TACE alone at the Eastern Hepatobiliary Surgery Hospital, The First Hospital of Jiaxing Affiliated Hospital of Jiaxing University and The First Affiliated Hospital of Wenzhou Medical University were retrospectively reviewed. The diagnosis of recurrent HCC was confirmed by dynamic CT and/or MRI according to the European Society for Medical Oncology. The inclusion criteria were as follows:(1) aged between 18‐ and 80 years; (2) suffered from the first recurrence after curative resection with a pathological examination; (3) had two to three lesions where at least one was >3 cm or more than three lesions, and in up‐to‐seven criteria; (4) Child‐Pugh stage A or B; (5) ECOG Score 0 or 1. And patients were excluded if they (1) received other treatments; (2) had macrovascular invasion or other organ metastasis; (3) accompanied with severe medical comorbidities including pulmonary, cardiac, or renal dysfunction; (4) had active or history of immunodeficiency or autoimmune disease; (5) lost to follow‐up .

This retrospective study was conducted in accordance with the Declaration of Helsinki. Ethical approval was obtained from the Institutional Ethics Committees of Eastern Hepatobiliary Surgery Hospital, The First Hospital of Jiaxing Affiliated Hospital of Jiaxing University, and The First Affiliated Hospital of Wenzhou Medical University. Informed consent was obtained from each participating patient.

### 2.2 Treatment

Our multidisciplinary team explained the recommended and alternative treatment options to the patients, including advantages and disadvantages of different treatment methods, potential treatment‐related side effects, possible treatment outcomes, and costs. The patients made the final treatment choice and signed informed consent. The method of TACE procedure had been described in our previous study.^18^, ^19^ Repeat TACE was based on the “on‐demand” principle. The initial use of lenvatinb or lenvatinib plus PD‐1 inhibitor was within 7 days after the first TACE treatment. Lenvatinib dose was 8 mg (<60 kg) or 12 mg (≥60 kg) once daily based on body weight. PD‐1 inhibitors were administered intravenously at standard doses (Sintilimab, 200 mg/3 weeks; Toripalimab, 240 mg/3 weeks; Camrelizumab, 200 mg/3 weeks). Treatment‐related adverse events (AEs) were assessed according to the Common Terminology Criteria for Adverse Events (CTCAE, version 5.0). Dosage reduction was allowed if a Grade 3 or 4 AE occurred. Dose adjustments were performed according to the drug's instructions. The subsequent treatment strategies were determined according to the full discussion of the multidisciplinary team and patients.

### 2.3 Follow‐up and assessment

Routine follow‐up was conducted every 6–8 weeks after initial TACE. The last follow‐up time for this study ended on February 25, 2022. The follow‐up items included medical history taking, physical examination, laboratory tests (blood count, liver function, alpha‐fetoprotein (AFP) and des‐gamma‐carboxy prothrombin (DCP)), contrast‐enhanced CT or MRI.

The primary endpoint was PFS, defined as the time from first TACE to TACE failure/refractoriness, untreatable progression, or death from any cause. The secondary endpoint was OS, which was specified as the time from the first TACE to death from any reason or the last follow‐up visit. Tumor responses were classified into complete response (CR), partial response (PR), stable disease (SD), and progressive disease (PD)according to the mRECIST standard.^20^ The ORR was defined as the sum of the CR and PR. The disease control rate (DCR) was defined as the sum of the CR, PR, and SD.

### 2.4 Statistical analysis

Categorical variables are expressed as frequency (%). Continuous variables with normal and non‐normal distributions are expressed as mean ± standard deviation and median (interquartile range), respectively. Pearson's chi‐squared test or Fisher's exact test was used for the comparison of categorical variables. The *t*‐test and non‐parametric test were used to analyze continuous variables. Survival curves were estimated and differences were analyzed using the Kaplan–Meier method and log‐rank test. The comparison of OS and PFS was further analyzed by Bonferroni correction. Independent prognostic factors were identified using univariate and multivariate COX regression analysis. All variables (*p* < 0.1) in the univariate analysis were included in the multivariate Cox regression analysis. Statistical analysis was based on a two‐tailed hypothesis test. *p* < 0.05 was considered statistically significant. The data were analyzed by SPSS version 26.0 (IBM Corp) and GraphPad Prism (version 7.0; GraphPad, Inc.).

## 3 RESULTS

### 3.1 Patient characteristics

**FIGURE 1:**
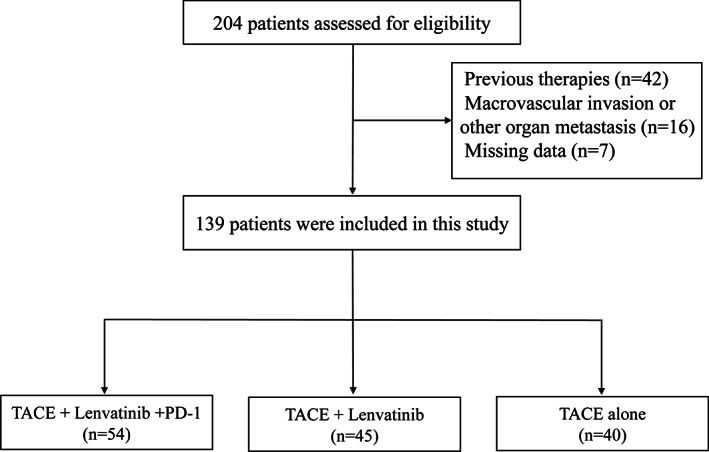
The flowchart to select eligible unresectable recurrent HCC patients.

Between January 2019 and December 2020, 204 patients with unresectable recurrent HCC who received T‐L‐P, T‐L, or TACE alone treatment were screened, of whom 65 patients were excluded (Figure 1). Ultimately, a total of 139 patients were included in the study, including 54 in the T‐L‐P group, 45 in the T‐L group, and 40 in the TACE alone group. Collection of all baseline characteristics was performed prior to the first TACE treatment (Table 1), and there were no significant differences among the three groups.

**TABLE 1:** Baseline characteristics of study patients.

Characteristics	T‐L‐P group (*n* = 54)	T‐L group (*n* = 45)	TACE alone (*n* = 40)	*p‐*value
Age (year)				0.142
Median ± SD	57.0 ± 9.9	60.8 ± 9.4	58.6 ± 9.4	
Range	35–79	43–80	40–74	
Sex				0.684
Male	49(90.7)	43 (95.6)	38 (95.0)	
Female	5(9.3)	2 (4.4)	2(5.0)	
Chronic hepatitis B				1.000
Yes	52 (96.3)	43 (95.6)	39(97.5)	
No	2 (3.7)	2 (4.4)	1(2.5)	
Hepatic cirrhosis				0.708
Yes	20 (37.0)	13 (28.9)	14(35.0)	
No	34 (63.0)	32 (71.1)	26(65.0)	
AFP (ng/mL)				0.455
≥400	14 (25.9)	7 (15.6)	8(20.0)	
<400	40 (74.1)	38 (84.4)	32(80.0)	
DCP (mAU/mL)				0.482
≥2050	12 (22.2)	9 (20.0)	5(12.5)	
<2050	42 (77.8)	36 (80.0)	35(87.5)	
MVI				0.465
MVI (−)	27 (50.0)	28 (62.2)	23(57.5)	
MVI (+)	27 (50.0)	17 (37.8)	17(42.5)	
Tumor size (cm)				0.134
≥2	28 (51.9)	21 (46.7)	15(37.5)	
<2	26 (48.1)	41 (53.3)	25(62.5)	
Recurrence interval (year)				0.528
<2	44 (81.5)	33 (73.3)	33(82.5)	
≥2	10 (18.5)	12 (26.7)	7(17.5)	
TB (μmol/L)				0.129
Median ± SD	22.3 ± 14.2	18.2 ± 10.5	18.1 ± 8.8	
Range	6.0–69.2	7.1–73.2	7.5–56.4	
ALB (g/L)				0.378
Median ± SD	40.3 ± 6.4	41.3 ± 7.4	42.2 ± 5.6	
Range	16.0–55.0	17.0–67.4	21.0–67.4	
ALT (U/L)				0.401
Median ± SD	45.1 ± 24.5	38.7 ± 24.9	44.0 ± 23.9	
Range	8.0–94.0	9.0–107.0	9.0–96.0	
AST (U/L)				0.940
Median ± SD	45.5 ± 20.6	46.9 ± 28.4	47.0 ± 24.6	
Range	11.0–86.0	14.0–130.0	10.0–121.0	
PT (sec)				0.274
Median ± SD	12.0 ± 1.1	11.8 ± 1.0	11.7 ± 0.74	
Range	10.2–15.1	10.4–14.3	10.6–13.7	
ALBI grade				0.689
1	28 (51.9)	28 (62.2)	25 (62.5)	
2	25 (46.3)	16 (35.6)	15 (37.5)	
3	1 (1.9)	1 (2.2)	0 (0)	
Child–Pugh				0.663
A (5–6)	49 (90.7)	42 (93.3)	35 (87.5)	
B (7–8)	5 (9.3)	3 (6.7)	5 (12.5)	
ECOG (performance status)				0.522
0	46 (85.2)	41 (91.1)	33 (82.5)	
1	8 (14.8)	4 (8.9)	7(17.5)	
HBV‐DNA (IU/mL)				0.302
≥50	6 (11.1)	4 (8.9)	1(2.5)	
<50	48 (88.9)	41 (91.1)	39(97.5)	
PD‐1 categories				‐
Toripalimab	30 (55.6)	‐	‐	
Sintilimab	21 (38.8)	‐	‐	
Camrelizumab	3 (5.6)	‐	‐	

Abbreviations: AFP, alpha‐fetoprotein concentration; DCP, Des‐gamma‐carboxy prothrombin; ALT, alanine aminotransferase; AST, aspartate aminotransferase; MVI, microvascular invasion; PT, prothrombin time; TACE, transarterial chemoembolization; TB, total bilirubin; ALB, albumin; T‐L, transarterial chemoembolization plus lenvatinib; T‐L‐P, transarterial chemoembolization combined with lenvatinib plus programmed cell death protein‐1 inhibitors.

### 3.2 Efficacy

**FIGURE 2:**
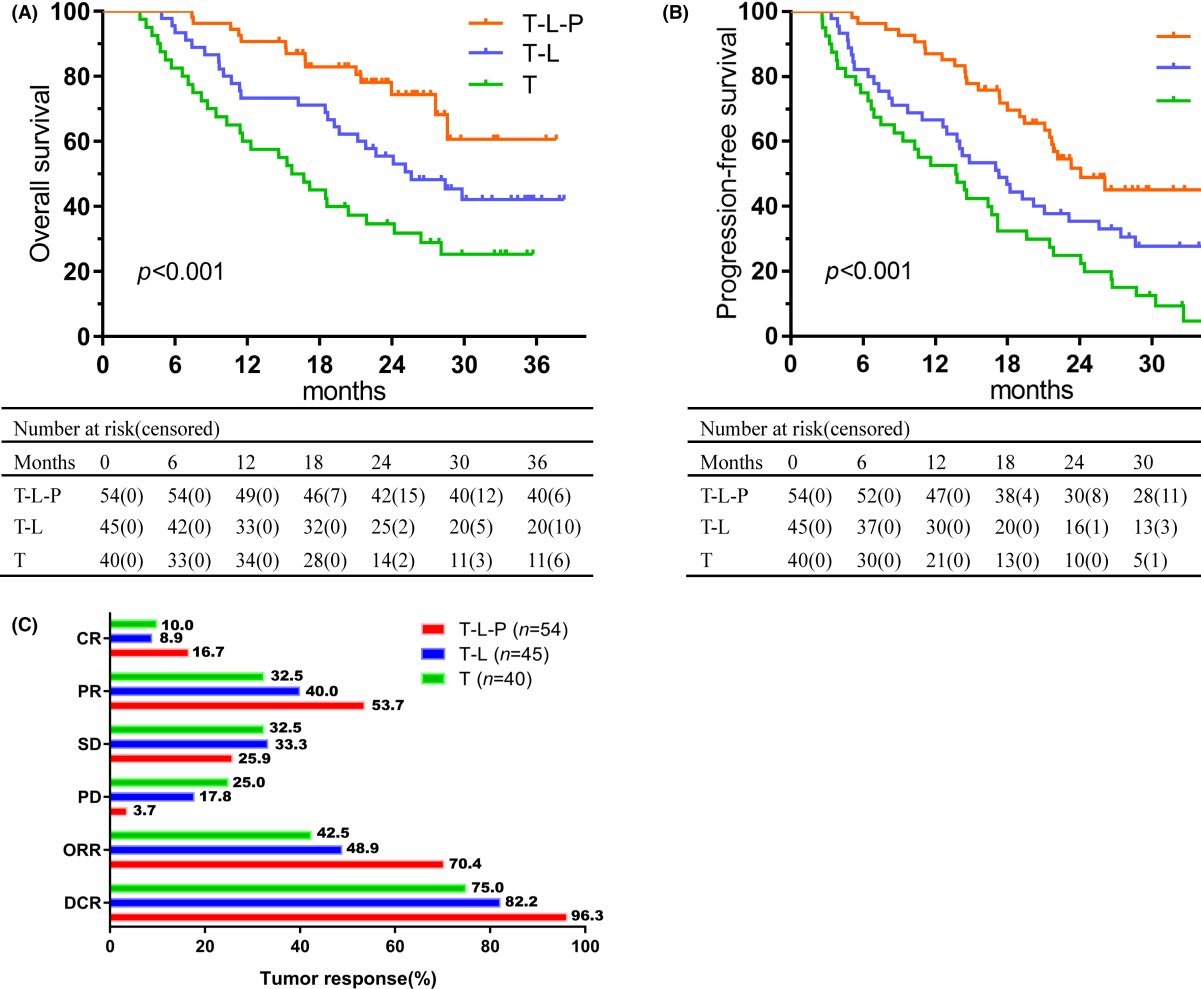
Overall survival (A) and progression‐free survival (B) were analyzed by Kaplan–Meier method according to treatment groups, and tumor responses (C) in each cohort.

The median follow‐up time for the T‐L‐P, T‐L, and TACE alone groups were 23.7, 33.9, and 32.5 months, respectively. Statistically significant differences in median OS and median PFS were observed among the three groups. The median OS in the T‐L‐P, T‐L, and TACE alone groups were not reached, 25.6 and 15.7 months, respectively (*p* < 0.001; Figure 2A). The median PFS in the T‐L‐P, T‐L, and TACE alone groups were 24.1, 17.3, and 13.7 months, respectively (*p* < 0.001; Figure 2B). Significant differences were identified for both OS and PFS after Bonferroni correction (*α* = 0.0167). The T‐L‐P group had significantly prolonged median OS and median PFS than the TACE alone group (*p* < 0.001and *p* < 0.001, respectively). The *p*‐values for median OS in the T‐L‐P group versus the T‐L group and the T‐L group versus the TACE alone group were 0.026 and 0.033, respectively. The *p*‐values for median PFS in the T‐L‐P group versus the T‐L group and the T‐L group versus the TACE alone group were 0.022 and 0.037, respectively.

Assessment of tumor response was based on the mRECIST criteria (Figure 2C; Table S1). Both best ORR and DCR were higher in the T‐L‐P group. More specifically, the best ORR in the T‐L‐P, T‐L, and TACE alone groups were 70.4%, 48.9%, and 42.5%, respectively. The best DCR in the T‐L‐P, T‐L, and TACE alone groups were 100.0%, 97.8%, and 87.5%, respectively.

### 3.3 Prognostic factors analysis

Univariate and multivariate cox regression analysis were adopted to assess the risk factors for OS and PFS (Table 2). Hepatic cirrhosis (HR, 0.487; 95% CI, 0.276–0.857; *p* = 0.013) and anti‐PD‐1 treatment (HR, 0.239; 95% CI, 0.126–0.455; *p* < 0.001) were significant independent predictors for OS. Furthermore, MVI (HR, 1.610; 95% CI, 1.068–2.429; *p* = 0.023), DCP (HR, 1.663; 95% CI, 1.011–2.737; *p* = 0.045), and anti‐PD‐1 treatment (HR, 0.314; 95% CI, 0.188–0.523; *p* < 0.001) were identified as independent risk factors for PFS.

**TABLE 2:** Univariate and multivariate cox regression analyses for PFS and OS.

Factor	Progression‐free survival						Overall survival					
	Univariate analysis			Multivariate analysis			Univariate analysis			Multivariate analysis		
	*p* Value	HR	95% CI	*p* Value	HR	95% CI	*p* Value	HR	95% CI	*p* Value	HR	95% CI
Age	0.366	0.990	(0.968,1.012)				0.227	0.984	(0.959,1.010)			
Sex (male vs. female)	0.955	0.978	(0.450,2.125)				0.711	0.853	(0.367,1.979)			
Chronic hepatitis B (negative vs. positive)	0.314	2.058	(0.505,8.397)				0.706	1.312	(0.320,5.386)			
Hepatic cirrhosis (negative vs. positive)	0.133	0.715	(0.462,1.107)				0.013	0.487	(0.276,0.857)	0.013	0.487	(0.276,0.857)
AFP	0.055	1.620	(0.990,2.650)				0.096	1.639	(0.917,2.931)			
DCP	0.087	1.536	(0.939,2.511)	0.045	1.663	(1.011,2.737)	0.328	1.345	(0.743,2.434)			
MVI (negative vs. positive)	0.041	1.530	(1.017,2.302)	0.023	1.610	(1.068,2.429)	0.114	1.472	(0.911,2.376)			
Tumor size (<2 cm vs.≥2 cm)	0.815	1.049	(0.700,1.574)				0.515	0.851	(0.524,1.383)			
Recurrence time (< 2 years vs. ≥2 years)	0.120	0.662	(0.394,1.113)				0.306	0.728	(0.396,1.337)			
TB	0.573	0.995	(0.976,1.013)				0.256	0.985	(0.960,1.011)			
ALB	0.109	0.975	(0.946,1.006)				0.355	0.982	(0.946,1.020)			
ALT	0.257	0.995	(0.987,1.004)				0.378	0.995	(0.986,1.006)			
AST	0.803	1.001	(0.993,1.010)				0.514	1.003	(0.993,1.013)			
PT	0.116	1.174	(0.961,1.433)				0.102	1.222	(0.961,1.553)			
Child–Pugh (A vs. B)	0.357	0.722	(0.361,1.444)				0.382	0.686	(0.294,1.597)			
ECOG (0 vs. 1)	0.382	1.281	(0.735,2.230)				0.829	1.081	(0.533,2.194)			
HBV‐DNA	0.417	1.355	(0.651,2.823)				0.171	1.743	(0.787,3.863)			
Anti‐PD‐1 treatment												
TACE alone	0.000			0.000			0.000			0.000		
T‐L T‐L‐P	0.036 <0.001	0.603 0.346	(0.375,0.969) (0.209,0.572)	0.046 <0.001	0.616 0.314	(0.382.0.991) (0.188,0.523)	0.031 <0.001	0.554 0.261	(0.324,0.947) (0.138,0.494)	0.012 <0.001	0.498 0.239	(0.290,0.857) (0.126,0.455)

Abbreviations: AFP, alpha‐fetoprotein concentration; ALB, albumin; ALT, alanine aminotransferase; AST, aspartate aminotransferase; DCP, Des‐gamma‐carboxy prothrombin; HR, hazard rate; CI, confidence interval; MVI, microvascular invasion; PD‐1, programmed cell death protein‐1; PT, prothrombin time; TB, total bilirubin.

**FIGURE 3:**
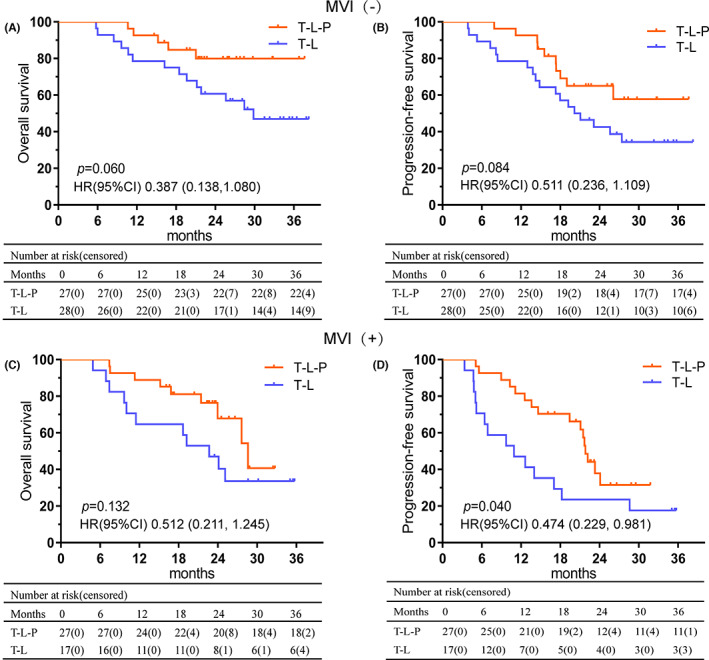
Kaplan–Meier analyses of overall survival (A) and progression‐free survival (B) according to combination treatments in MVI‐negative patients. Kaplan–Meier analyses of overall survival (C) and progression‐free survival (D) according to combination treatments in MVI‐positive patients.

Subgroup analyses according to the presence of MVI demonstrated that, when compared to T‐L, T‐L‐P provided a better PFS in MVI‐positive patients (HR, 0.474; 95% CI, 0.229–0.981; *p* < 0.001). However, there was no significant difference in OS in both the MVI‐positive and negative groups (Figure 3).

### 3.4 Safety analysis

In this study, there were no patient deaths due to treatment in the T‐L‐P, T‐L, and TACE alone groups, and the details of AEs were recorded (Table 3). All major AEs were treated symptomatically and patients recovered within a short time. Compared with TACE monotherapy, T‐L‐P and T‐L combination did not significantly increase the incidence of AEs for any grade except hypertension and decreased appetite. One patient in the TACE alone group suffered from Grade 3/4 AE, while 19 (35.2%) and 10 (22.2%) in the T‐L‐P and T‐L groups, respectively. Notably, there was no significant difference between the T‐L‐P and T‐L groups for Grade 3/4 AEs. The predominant Grade 3/4 AE in T‐L‐P (6 patients, 11.1%) and T‐L (4 patients, 8.9%) was hypertension.

**TABLE 3:** Treatment emergent adverse events.

Adverse events	Any grade, *n* (%)				Grade 3/4, *n* (%)			
	T‐L‐P (*n* = 54)	T‐L (*n* = 45)	T (*n* = 40)	*p*‐value	T‐L‐P (*n* = 54)	T‐L (*n* = 45)	T (*n* = 40)	*p‐*value
Elevated TB	8 (14.8)	10 (22.2)	7 (17.5)	0.844	1 (1.9)	0 (0)	0 (0)	1.000
Elevated AST	20 (37.0)	13 (28.9)	11 (27.5)	0.548	2 (3.7)	1 (2.2)	0 (0)	0.222
Elevated ALT	25 (46.3)	15 (33.3)	10 (25.0)	0.094	5 (9.3)	3 (6.7)	0 (0)	0.137
Decreased PLT	15 (27.8)	11 (24.4)	4 (10.0)	0.100	3 (5.6)	1 (2.2)	1 (2.5)	0.734
WBC decrease	7 (13.0)	5 (11.1)	4 (10.0)	0.945	0 (0)	0 (0)	0 (0)	–
Neutropenia	5 (9.3)	2 (4.4)	1 (2.5)	0.387	0 (0)	0 (0)	0 (0)	–
Decreased albumin	2 (3.7)	2 (4.4)	0 (0)	0.556	0 (0)	0 (0)	0 (0)	–
Hemoglobin decreased	3 (5.6)	1 (2.2)	0 (0)	0.461	0 (0)	0 (0)	0 (0)	–
Hypothyroidism	2 (3.7)	0 (0)	0 (0)	0.334	0 (0)	0 (0)	0 (0)	–
Hypertension	16 (29.6)	10 (22.2)	0 (0)	<0.001	6 (11.1)	4 (8.9)	0 (0)	0.068
Hand‐foot skin reaction	6 (11.1)	4 (8.9)	0 (0)	0.068	2 (3.7)	1 (2.2)	0 (0)	0.778
Decreased appetite	8 (14.8)	4 (8.9)	0 (0)	0.027	0 (0)	0 (0)	0 (0)	–
Fever	10 (18.5)	6 (13.3)	7 (17.5)	0.773	0 (0)	0 (0)	0 (0)	–
Fatigue	11 (20.4)	7 (15.6)	5 (12.5)	0.583	0 (0)	0 (0)	0 (0)	–
Abdominal pain	6 (11.1)	7 (15.6)	9 (22.5)	0.326	0 (0)	0 (0)	0 (0)	–
Vomiting	9 (16.7)	4 (8.9)	4 (10.0)	0.519	0 (0)	0 (0)	0 (0)	–
Diarrhea	6 (11.1)	5 (11.1)	3 (7.5)	0.880	0 (0)	0 (0)	0 (0)	–
Dysphonia	3 (5.6)	2 (4.4)	0 (0)	0.443	0 (0)	0 (0)	0 (0)	–
proteinuria	2 (3.7)	2 (4.4)	0 (0)	0.556	0 (0)	0 (0)	0 (0)	–

Abbreviations: AEs, adverse events; ALT, alanine aminotransferase; AST, aspartate transaminase; PLT, platelet; T, transarterial chemoembolization; TB, total bilirubin; T‐L, transcatheter arterial chemoembolization plus lenvatinib; T‐L‐P, transcatheter arterial chemoembolization combined with lenvatinib plus programmed cell death protein‐1 inhibitors; WBC, white blood cell.

### 3.5 Subsequent treatment

Eleven patients received subsequent therapy after 27 patients developed disease progression in the T‐L‐P group, while 12 patients received subsequent therapy after 31 patients in the T‐L group experienced disease progression in the T‐L group. Most patients choose a combination of RFA and TACE or a combination of locoregional therapy and immunotherapy in the T‐L‐P group. In the T‐L group, four of the patients with progressive disease were added to PD‐1 inhibitors and four patients opted for a combination of local therapy with RFA and TACE (Table S2).

## 4 DISCUSSION

Despite various treatment strategies for unresectable recurrent HCC, the optimal regimen has not reached a consensus.^21^, ^22^ Recently, several types of research have demonstrated the efficacy of different double combination treatments for recurrent HCC,^11^, ^23^, ^24^, ^25^ but no study has evaluated whether the combination of TACE, lenvatinib, and PD‐1 treatment could further improve the prognosis. As far as we know, this retrospective study is the first one to investigate the survival benefit and safety of T‐L‐P combination treatment in patients with unresectable recurrent HCC.

The present study showed both T‐L‐P and T‐L contributed to longer PFS and OS when compared with TACE monotherapy in unresectable recurrent HCC, and T‐L‐P conferred more benefits than T‐L. The survival benefit may be due to higher ORR and DCR. As a common regimen for recurrent HCC, TACE possesses the ischemic and cytotoxic antitumor function which may induce a local hypoxic environment and promotes neovascularization.^26^, ^27^, ^28^ The efficacy of TACE alone in recurrent intermediate‐stage HCC patients was limited with 44.1% ORR and 76.6% DCR, and the median OS was 14.43 months.^29^ Vascular endothelial growth factor (VEGF) inhibitor could reverse the high angiogenic factor secretion and suppress the remnant tumor recurrence and metastasis.^30^, ^31^, ^32^, ^33^ Thus, some studies evaluated the efficacy of VEGF inhibitors for recurrent HCC treated with TACE and confirmed this regimen could provide long‐term benefits.^11^, ^12^, ^34^ More notably, a prospective randomized study found the combination of TACE plus lenvatinib achieved a higher ORR and median time to progression than the combination of TACE plus sorafenib for advanced HCC.^35^ Additionally, both TACE and lenvatinib could alter the tumor immune microenvironment.^36^, ^37^, ^38^ The expression of PD‐1/PD‐L1 was significantly increased in patients treated with TACE by immunohistochemical analysis, and lenvatinib could reverse the increase of immunosuppressive cell types, such as tumor‐associated macrophages, myeloid‐derived suppressor cells, regulatory T cells, and so on. Therefore, T‐L‐P might be a synergistic regimen and exhibited better efficacy for unresectable recurrent HCC.^16^, ^39^

Furthermore, in addition, our study showed that the presence of MVI was an independent risk factor for PFS. Of note, in subgroup analyses, T‐L‐P treatment significantly prolonged PFS but not OS in MVI‐positive patients when compared with T‐L treatment, while neither PFS nor OS was significantly different in the comparison between these two combination treatments. Several possible explanations might be contributed to the results. First, no significant difference might be caused by the limited number of patients. Second, various subsequent treatments after the progression may also influence the OS of patients. Besides, MVI‐positive lesions indicated aggressive biological behavior, and the up‐regulation of PD‐1/PD‐L1 was found in some studies,^40^, ^41^ so the concurrent use of PD‐1 may have a positive effect on delaying the disease progression in MVI‐positive patients.

In addition, the safety of the combined treatment was investigated. Triple or dual therapy may inevitably increase the incidence of adverse events, and the most common adverse event was impaired liver function. Of note, when compared with TACE monotherapy, the incidence of AEs in both the T‐L‐P group and the T‐L group was no significant difference except for hypertension and decreased appetite. More patients occurred hypertension might result from the simultaneous effect on angiogenesis by TACE and lenvatinb, and appetite decreased mostly due to the more toxicities brought by the combination treatment.^42^, ^43^ Furthermore, the combination treatments would not increase the occurrence of Grade 3 or 4 AEs when compared with TACE monotherapy, and the most frequent Grade 3 or 4 AEs were elevated ALT and AST, hypertension, and decreased PLT.^44^, ^45^ Therefore, both the triple and double combination treatments would not increase the risk for unmanageable AEs, and clinicians should pay more attention to the drug toxicity and liver function after the application.

There are several limitations to this study. First, this study was retrospective and the relatively small number of patients may lead to selection bias. Second, the cost and availability of treatment may influence patients' choice of treatment strategy, which may also introduce selection bias. Third, the period of follow‐up was relatively short. Therefore, prospective multicenter clinical trials are necessary to validate our results.

## 5 CONCLUSION

In summary, this study is the first one to evaluate the efficacy and safety of the T‐L‐P regimen in the treatment of unresectable recurrent HCC, and the results showed that this combination was safe and superior to T‐L or TACE alone in improving survival for unresectable recurrent HCC patients.

## AUTHOR CONTRIBUTIONS

**Wei‐Jun Wang:** Conceptualization (equal); formal analysis (lead); methodology (lead); writing – original draft (lead); writing – review and editing (lead). **Zonghan Liu:** Conceptualization (equal); data curation (equal); writing – original draft (equal). **Kang Wang:** Conceptualization (equal). **Hong‐Ming Yu:** Data curation (equal). **Yu‐Qiang Cheng:** Data curation (equal). **Yan‐Jun Xiang:** Data curation (equal); formal analysis (equal). **Jin‐Kai Feng:** Data curation (equal); formal analysis (equal). **Li‐Ping Zhou:** Data curation (equal); investigation (equal). **Hong‐Kun Zhou:** Data curation (equal). **Wei‐Wei Pan:** Data curation (equal). **Wei‐Xing Guo:** Data curation (equal). **Jie Shi:** Data curation (equal). **Shuqun Cheng:** Conceptualization (equal); funding acquisition (lead); resources (lead); supervision (lead); validation (lead); writing – review and editing (equal).

## FUNDING INFORMATION

This work was supported by the Clinical Research Plan of SHDC (No. SHDC2020CR1004A), the State Key Program of National Natural Science Foundation of China (No: 81730097), the National Natural Science Foundation of China (No: 82072618 and No: 81770630), the Science and Technology Commission Foundation of Shanghai Municipality (No: 19411967300), and the National Key Research and Development Program of China (2022YFC2503700).

## CONFLICT OF INTEREST STATEMENT

The authors who participate in this study have no conflicts of interest to declare.

## ETHICS STATEMENT

The study conducted in accordance with the Declaration of Helsinki and the International Conference on Harmonization guidelines for Good Clinical Practice and approved by the Eastern Hepatobiliary Surgery Hospital. Ethical approvals were obtained from the ethical review committee of Eastern Hepatobiliary Hospital of the main center. Written informed consentobtained for participation in this study.

## Supporting information


Table S1.

Table S2.

## Data Availability

The data that support the findings of this study were available from the corresponding author upon reasonable request.
