# Supplementary material for: Efficacy and safety of TACE combined with lenvatinib and PD‐1 inhibitors for unresectable recurrent HCC: A multicenter, retrospective study
Source: Cancer Med. 2023 Mar 31;12(10):11513–24. doi: 10.1002/cam4.5880 (PMC10242311; doi:10.1002/cam4.5880)
Supplement: Supplementary file 1 — Table S1. [file CAM4-12-11513-s002.docx]

| **Table S1.** Summary of tumor responses in three groups (mRECIST). | | | |
| --- | --- | --- | --- |
| Variables, n (%) | T-L-P (n=54) | T-L (n=45) | TACE alone (n=40) |
| CR | 9 (16.7) | 4 (8.9) | 4(10.0) |
| PR | 29 (53.7) | 18 (40.0) | 13(32.5) |
| SD | 16 (29.6) | 22 (48.9) | 18(45.0) |
| PD | 0 (0) | 1 (2.2) | 5(12.5) |
| ORR | 38 (70.4) | 22(48.9) | 17(42.5) |
| DCR | 54(100.0) | 44(97.8) | 35(87.5) |

*T-L-P, transarterial chemoembolization combined with lenvatinib plus programmed cell death protein-1 inhibitors; T-L, transarterial chemoembolization plus lenvatinib; TACE, transcarterial chemoembolization;* *CR, complete response; PR, partial response; SD, stable disease; PD, progress disease; ORR, objective response rate; DCR, disease control rate.*
