# Supplementary material for: Efficacy and safety of TACE combined with lenvatinib and PD‐1 inhibitors for unresectable recurrent HCC: A multicenter, retrospective study
Source: Cancer Med. 2023 Mar 31;12(10):11513–24. doi: 10.1002/cam4.5880 (PMC10242311; doi:10.1002/cam4.5880)
Supplement: Supplementary file 2 — Table S2. [file CAM4-12-11513-s001.docx]

**Table S2.** Subsequent treatment

|  | TACE+Lenvatinib+PD-1 (n=26) | TACE +Lenvatinib (n=32) | TACE alone  (n=37) |
| --- | --- | --- | --- |
| Accepted subsequent treatments | 11 | 15 | 17 |
| Radiotherapy +T+A | 4 | 2 | 0 |
| Ablation +TACE | 3 | 4 | 3 |
| TACE+PD-1 | 0 | 1 | 2 |
| TACE+T+A | 1 | 1 | 0 |
| TACE | 0 | 0 | 6 |
| TACE +Lenvatinib | 0 | 3 | 5 |
| TACE+Lenvatinib+PD-1 | 2 | 4 | 1 |
| Liver Transplantation | 1 | 0 | 0 |
| Best Supportive Care | 15 | 17 | 20 |

*TACE, transarterial chemoembolization; PD-1, programmed cell death-1 inhibitor; T+A, atezolizumab+ bevacizumab;*
